# Comparison of serum systemic inflammatory biomarkers in bone-milling robotic-assisted total knee arthroplasty and conventional total knee arthroplasty: a prospective randomized controlled trial

**DOI:** 10.1186/s43019-026-00309-5

**Published:** 2026-02-19

**Authors:** Peeranut Jittangtrong, Natthapong Hongku, Satit Thiengwittayaporn

**Affiliations:** Department of Orthopaedics, Faculty of Medicine Vajira Hospital, Navamindradhiraj University, 681 Samsen Rd, Dusit, Bangkok, 10300 Thailand

**Keywords:** Bone-milling robotic-assisted total knee arthroplasty, Conventional total knee arthroplasty, Serum systemic inflammatory biomarkers, Radiographic alignments

## Abstract

**Background:**

Robotic-assisted total knee arthroplasty (RA-TKA) aims to improve surgical accuracy and reduce soft-tissue trauma. The bone-milling technique may further decrease mechanical stress during bone preparation. This study primarily compared systemic inflammatory biomarkers between bone-milling RA-TKA and conventional TKA (C-TKA), with secondary assessments of perioperative parameters, radiographic alignment, and early postoperative outcomes.

**Methods:**

This prospective randomized controlled trial included 30 RA-TKAs and 30 C-TKAs performed between August 2023 and December 2024 in patients with Kellgren–Lawrence grade IV knee osteoarthritis. All RA-TKA procedures were conducted during the operating surgeon’s early learning phase with the robotic platform. Serum interleukin (IL)-6, erythrocyte sedimentation rate (ESR), C-reactive protein (CRP), creatine kinase (CK), and lactate dehydrogenase (LDH) were measured preoperatively and at 6 h, 1 day, 3 days, 2 weeks, and 6 weeks postoperatively. Perioperative variables, radiographic alignment, and 6-week Knee Society Score (KSS) and visual analog scale (VAS) pain scores were compared.

**Results:**

Postoperative inflammatory biomarkers did not differ significantly between groups at any time point, and the corresponding effect sizes were small, indicating minimal biological differences. Estimated blood loss was comparable (*p* = 0.753). RA-TKA demonstrated significantly improved postoperative mechanical alignment (mechanical axis [MA] deviation: 0.3 ± 2.4° versus 2.8 ± 3.4°; *p* = 0.002) but required longer tourniquet times (121.4 ± 15.3 min versus 95.0 ± 13.3 min; *p* < 0.001). Early functional outcomes were similar, with no significant differences in KSS (*p* = 0.114) or VAS pain scores at 6 weeks (*p* = 0.508).

**Conclusions:**

Bone-milling RA-TKA did not reduce systemic inflammatory responses compared with C-TKA, with small effect sizes confirming minimal biological differences. However, it provided superior radiographic alignment, while perioperative parameters and early postoperative recovery remained comparable except for longer tourniquet time.

## Introduction

Total knee arthroplasty (TKA) is a reliable and widely performed procedure for advanced knee osteoarthritis. Despite its well-established effectiveness, periarticular soft-tissue injury during surgery remains a concern, as it may adversely affect pain, early rehabilitation, and functional recovery [1, 2]. Minimizing surgical trauma has therefore become a central focus in efforts to enhance early postoperative recovery.

Robotic-assisted TKA (RA-TKA) has emerged as a technological advance designed to improve the precision of bone resection and soft-tissue balancing [3]. Jig-based RA-TKA has demonstrated superior accuracy in mechanical alignment compared with conventional TKA (C-TKA) [4–13], and several studies have reported reduced levels of systemic inflammatory biomarkers in the early postoperative period [13–17], suggesting decreased surgical trauma [16–19].

Robotic platforms incorporating functional or kinematic alignment planning may further reduce the need for soft-tissue releases. Additionally, the bone-milling technique, which uses a high-speed burr rather than a saw blade, has been introduced to potentially lessen periarticular tissue injury during bone preparation [18–21]. Despite this rationale, direct comparative data evaluating whether bone-milling RA-TKA reduces soft-tissue damage or improves early recovery remain insufficient. Although inflammatory biomarkers such as interleukin-6 (IL-6), C-reactive protein (CRP), erythrocyte sedimentation rate (ESR), creatine kinase (CK), and lactate dehydrogenase (LDH) are well established as indicators of tissue injury and systemic inflammation following TKA [22, 23], direct comparative evidence between bone-milling RA-TKA and C-TKA remains limited. The extent to which bone-milling RA-TKA can reduce soft-tissue damage and improve early postoperative recovery has not yet been clarified. In this study, early postoperative recovery was assessed using prespecified systemic biomarkers and validated clinical scores (Knee Society Score [KSS] and visual analog scale [VAS]), without the inclusion of additional recovery assessments. This study compared systemic inflammation, perioperative parameters, radiographic alignment, and early outcomes between bone-milling RA-TKA and C-TKA, hypothesizing that bone-milling RA-TKA would yield lower systemic biomarker levels, less soft-tissue injury, and better early recovery.

## Materials and methods

### Study design

The study was approved by the Institutional Review Board COA 099/2566 (study code: 057/66) and registered at the Clinical Trials Registry. Patients with knee osteoarthritis unresponsive to conservative treatment who underwent C-TKA or bone-milling RA-TKA (CORI, Smith & Nephew, Memphis, TN, USA) between August 2023 and December 2024 were included. Informed consent was obtained from all individual participants included in the study.

Patients aged 50–80 years with Kellgren–Lawrence grade IV knee osteoarthritis who did not respond to at least 6 months of conservative treatment were included. The exclusion criteria were autoimmune inflammatory arthritis, active malignancy, previous knee fractures, active infection (including coronavirus disease 2019 [COVID-19]), or American Society of Anesthesiologists (ASA) physical status class IV–VI.

Baseline data included age, sex, body mass index (BMI), operative side, ASA classification, comorbidities, hip–knee–ankle (HKA) angle, Knee Society Score (KSS), hematocrit, and inflammatory biomarkers (ESR, CRP, IL-6, CK, and LDH). Patients were randomized in blocks of four using sequentially numbered, opaque, sealed envelopes opened after induction of anesthesia. Outcome assessors remained blinded to group allocation throughout the follow-up period.

Estimated blood loss was calculated using the hemoglobin balance method, and total blood volume was estimated with the Gross equation [24].

### Surgical technique

At the time of study initiation, the operating surgeon had performed fewer than 50 RA-TKA cases with this platform. All procedures were conducted by the same surgeon under spinal anesthesia combined with an adductor canal block, following a standardized analgesic protocol for both groups. A midline skin incision and midvastus approach were used in all cases, and surgery was performed under tourniquet control. A fixed-bearing posterior-stabilized total knee prosthesis (Legion PS, Smith & Nephew), with patellar resurfacing, was implanted in all patients, and neutral mechanical alignment was targeted in both groups.

In the RA-TKA group, bone preparation was performed using a high-speed burr rather than an oscillating saw. Real-time intraoperative gap assessment was used to guide bone resection and component positioning, with the aim of achieving neutral mechanical alignment. In the C-TKA group, surgery followed standard mechanical alignment principles, including intramedullary femoral referencing, extramedullary tibial referencing, a predetermined valgus femoral cut of 5–7°, restoration of native tibial slope, and rotational alignment based on the posterior condylar axis and Whiteside’s line. Incision length, surgical approach, implant design, and postoperative protocols were identical between groups; the primary differences were the method of bone preparation and the use of robotic guidance with real-time gap assessment in the RA-TKA group.

Patellar resurfacing was performed using an onlay technique in all cases. All components were cemented, and 500 mg of intra-articular tranexamic acid was administered prior to capsule closure.

Postoperative management was identical in both groups, employing multimodal analgesia, early mobilization, and aspirin 81 mg twice daily for 14 days as venous thromboembolism prophylaxis. Quadriceps exercises and straight-leg raise commenced on postoperative day 1, followed by walker-assisted ambulation and twice-daily range of motion (ROM) exercises for 6 weeks. Most patients ambulated unaided within 2–3 weeks.

### Analysis of blood samples

Serum inflammatory biomarkers (ESR, CRP, IL-6, CK, and LDH) were measured preoperatively and at 6 h, 1 day, 3 days, 2 weeks, and 6 weeks postoperatively.

### Radiographic evaluation

Standard anteroposterior and lateral radiographs were obtained on postoperative day 1, and full-length weight-bearing HKA radiographs were obtained at 2 weeks. All patients stood without footwear, with the tibial tubercles facing forward, knees fully extended, and weight evenly distributed. Both lower limbs were imaged simultaneously.

### Clinical outcomes assessment

Estimated blood loss, tourniquet time, and early postoperative outcomes (Knee Society Score [KSS], visual analog scale [VAS] for pain) at 6 weeks postoperation were compared.

### Statistical analysis

Sample size was calculated from previous data comparing systemic inflammatory responses between RA-TKA and C-TKA [7]. Using CK levels at 6 h postoperatively (173.6 ± 51.1 IU/L versus 124.4 ± 57.4 IU/L), 60 patients (30:30) were required to achieve 90% power at *α* = 0.05 with a 10% dropout allowance. No correction for multiple comparisons was applied because inflammatory biomarkers were prespecified primary endpoints. Continuous variables were analyzed using *t*-tests and categorical variables using chi-squared tests, with significance set at *p* < 0.05. Analyses were performed using SPSS version 29.0 (IBM Corp., Armonk, NY, USA).

## Results

**Fig. 1:**
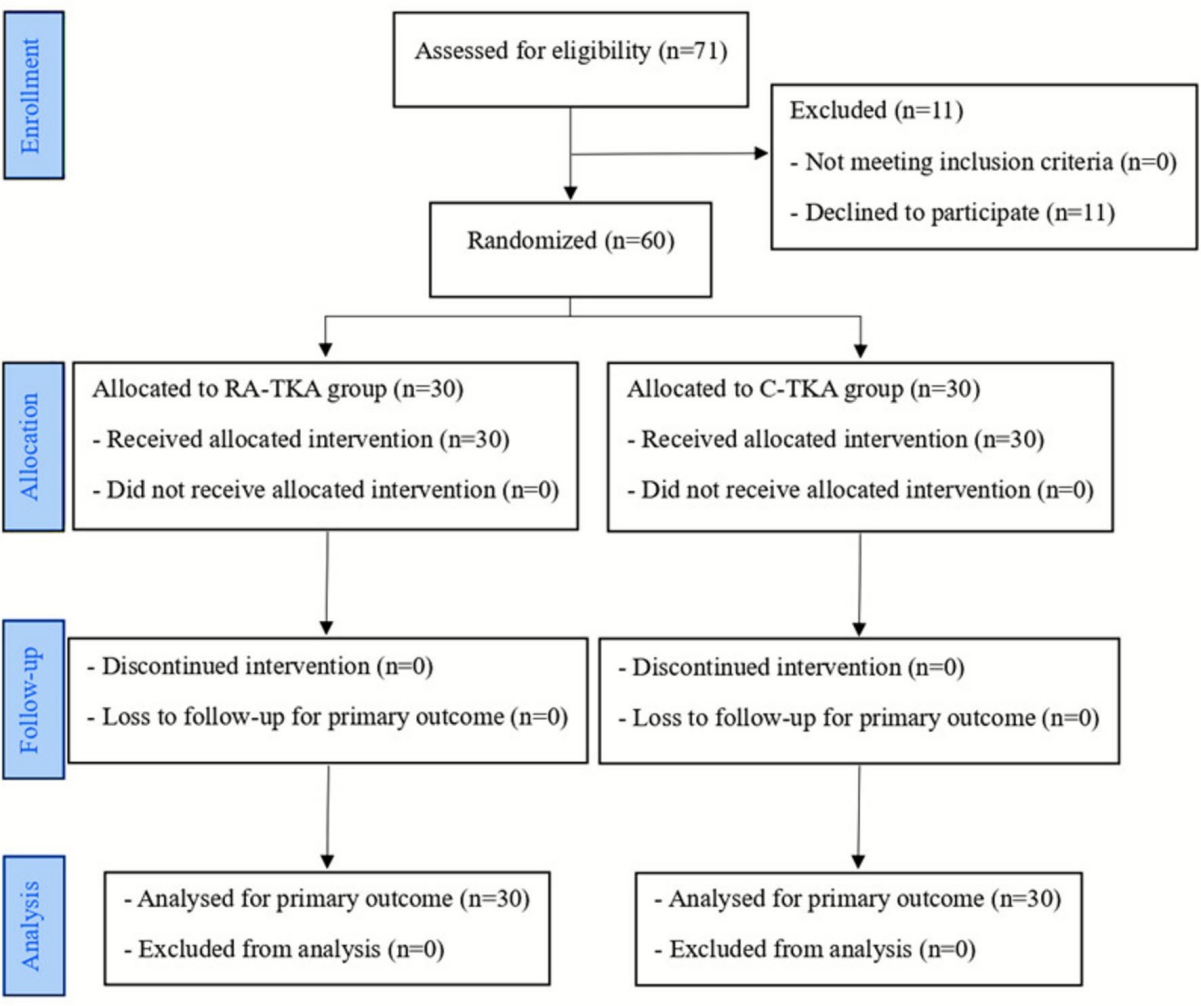
Consolidated Standard of Reporting Trials (CONSORT) flowchart of patients in this trial comparing bone-milling RA-TKA and C-TKA. *RA-TKA* robotic-assisted total knee arthroplasty, *C-TKA* conventional total knee arthroplasty

This prospective randomized controlled trial screened 71 patients with symptomatic primary knee osteoarthritis (Kellgren–Lawrence grade IV) who were scheduled to undergo primary unilateral TKA performed by a single experienced surgeon. Overall, 11 patients declined participation, leaving 60 eligible participants. These patients were then randomized into two groups: 30 assigned to C-TKA and 30 to bone-milling RA-TKA (Table 1; Fig. 1).

**Table 1:** Patient demographics, clinical scores, and radiographic parameters preoperatively

Characteristics	RA-TKA (*n* = 30)	C-TKA (*n* = 30)
Age (years)	68.3 ± 6.8	66.9 ± 6.4
Sex		
Male	25 (83)	23 (77)
Female	5 (17)	7 (23)
Weight (kg)	63.6 ± 10.3	66.3 ± 10.6
Height (cm)	155.5 ± 7.9	155.1 ± 7.9
BMI (kg/cm^2^)	26.2 ± 3.2	27.6 ± 4.0
Site		
Left	12 (40)	12 (40)
Right	18 (60)	18 (60)
ASA classification		
I	1 (3)	5 (17)
II	19 (63)	13 (43)
III	10 (33)	12 (40)
Mean preoperative Hct (%)	39.3 ± 3.3	39.8 ± 3.4)
Mean preoperative HKA (varus angle)	7.3 ± 5.7	8.7 ± 6.1
Preoperative VAS	6.8 ± 1.8	7.3 ± 1.8
Preoperative KSS score		
Knee score	32.4 ± 11.9	30.5 ± 11.3
Functional score	44.3 ± 14.6	40.6 ± 11.8
Patient satisfaction score	18.0 ± 5.8	18.6 ± 8.1
Patient expectation score	13.9 ± 1.3	14.2 ± 1.2

Values are presented as mean ± standard deviation or *n* (%)

*RA-TKA* robotic-assisted total knee arthroplasty, *C-TKA* conventional total knee arthroplasty, *BMI* body mass index, *ASA* American Society of Anesthesiologists, *Hct* Hematocrit, *HKA* hip–knee–ankle angle, *VAS* visual analog scale, *KSS* Knee Society Score

### Inflammatory biomarkers

**Fig. 2:**
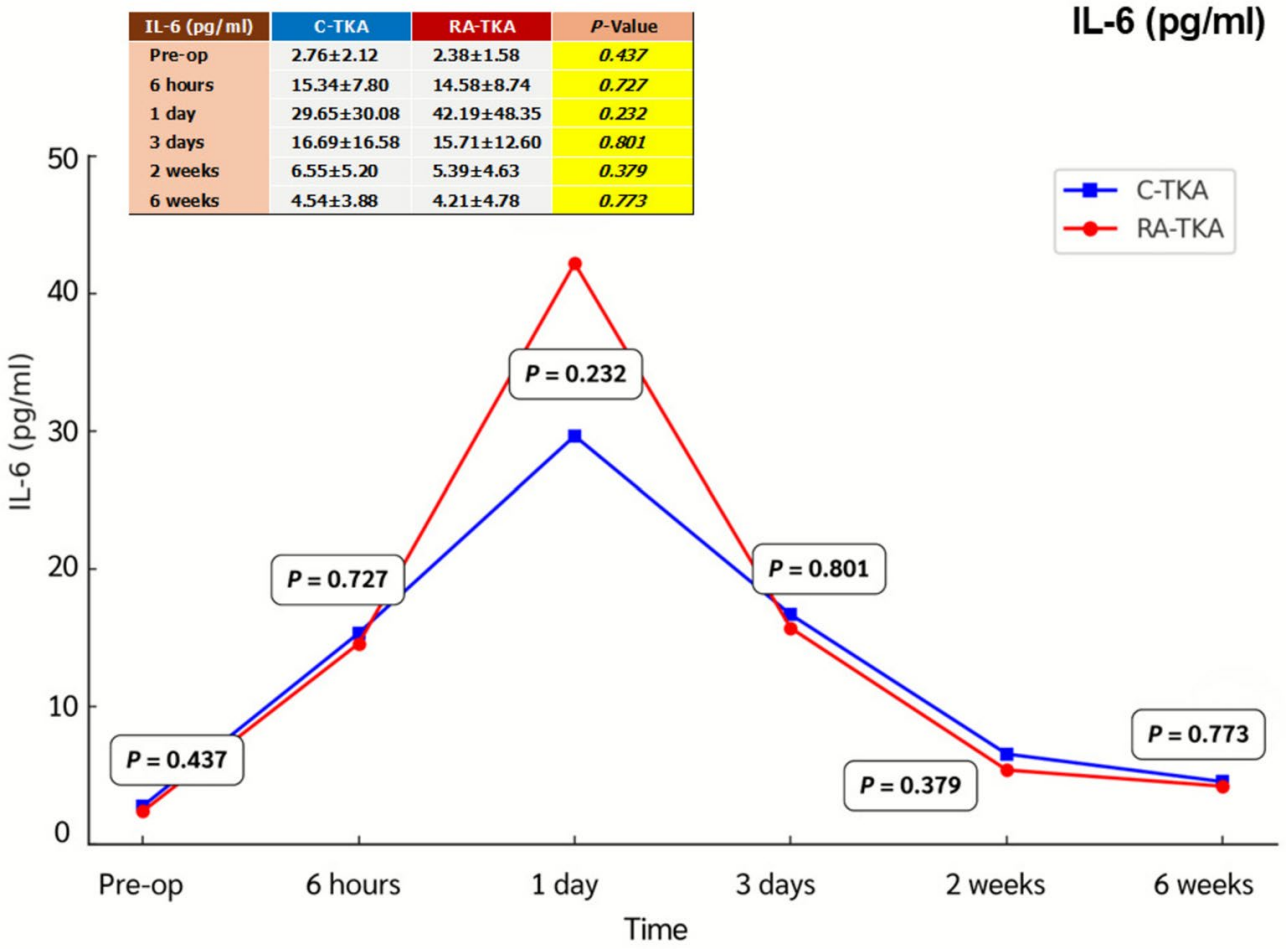
Comparison of systemic inflammatory biomarkers between C-TKA and RA-TKA in terms of interleukin-6 (IL-6) levels at preoperative and postoperative time intervals. *C-TKA* conventional total knee arthroplasty, *RA-TKA* robotic-assisted total knee arthroplasty

**Fig. 3:**
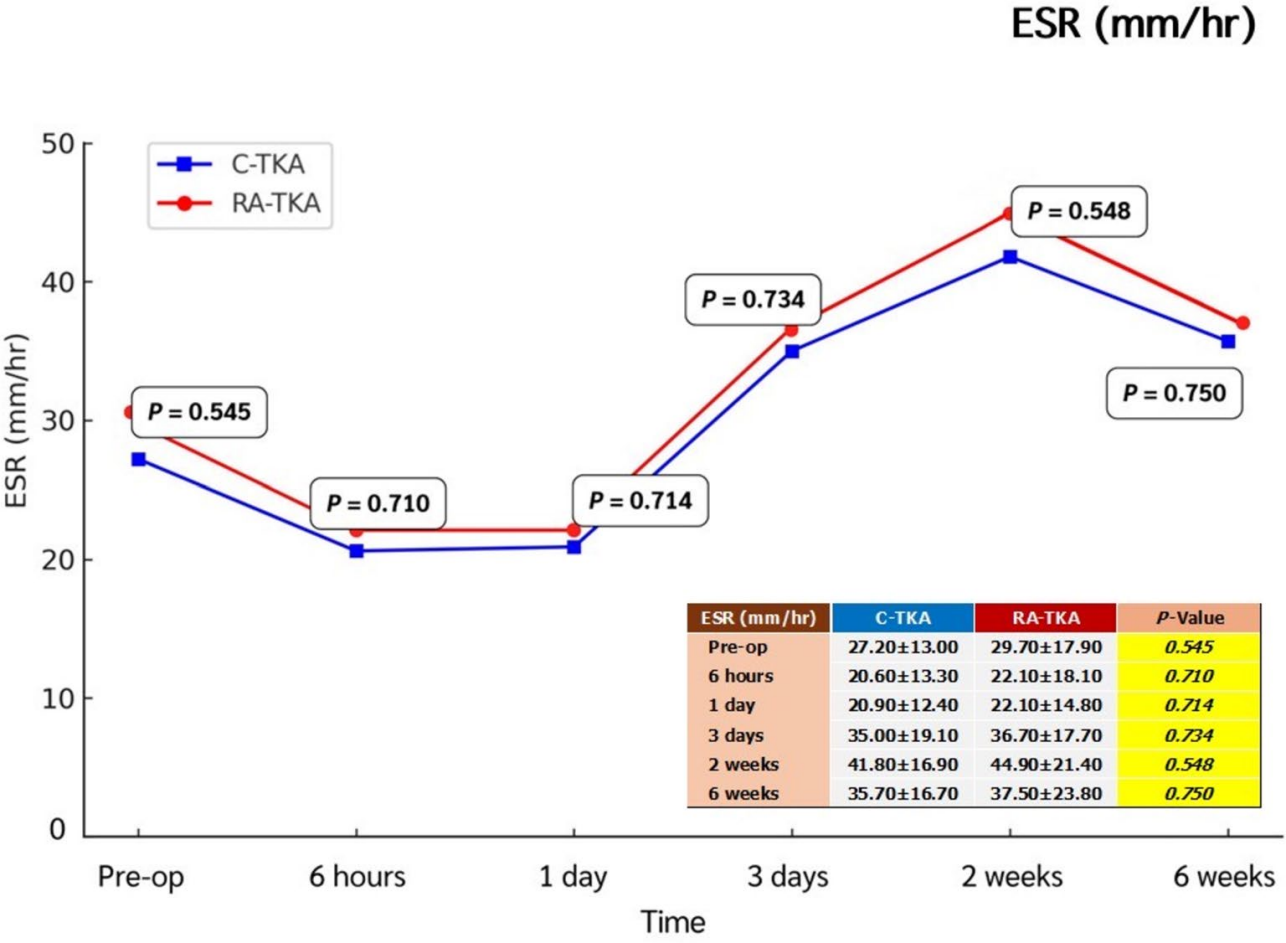
Comparison of systemic inflammatory biomarkers between C-TKA and RA-TKA in terms of erythrocyte sedimentation rate (ESR) levels at preoperative and postoperative time intervals. *C-TKA* conventional total knee arthroplasty, *RA-TKA* robotic-assisted total knee arthroplasty

**Fig. 4:**
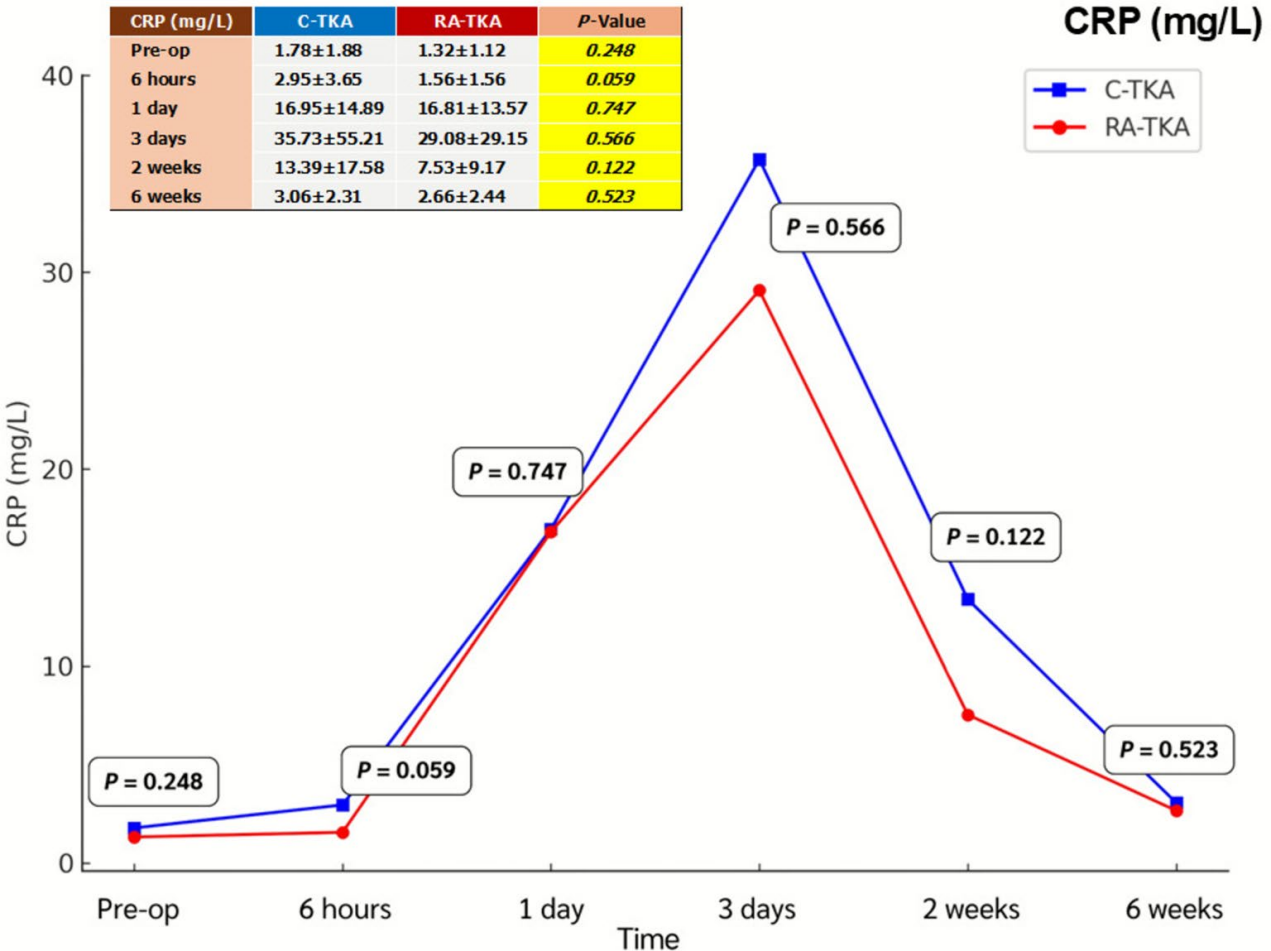
Comparison of systemic inflammatory biomarkers between C-TKA and RA-TKA in terms of C-reactive protein (CRP) levels at preoperative and postoperative time intervals. *C-TKA* conventional total knee arthroplasty, *RA-TKA* robotic-assisted total knee arthroplasty

**Fig. 5:**
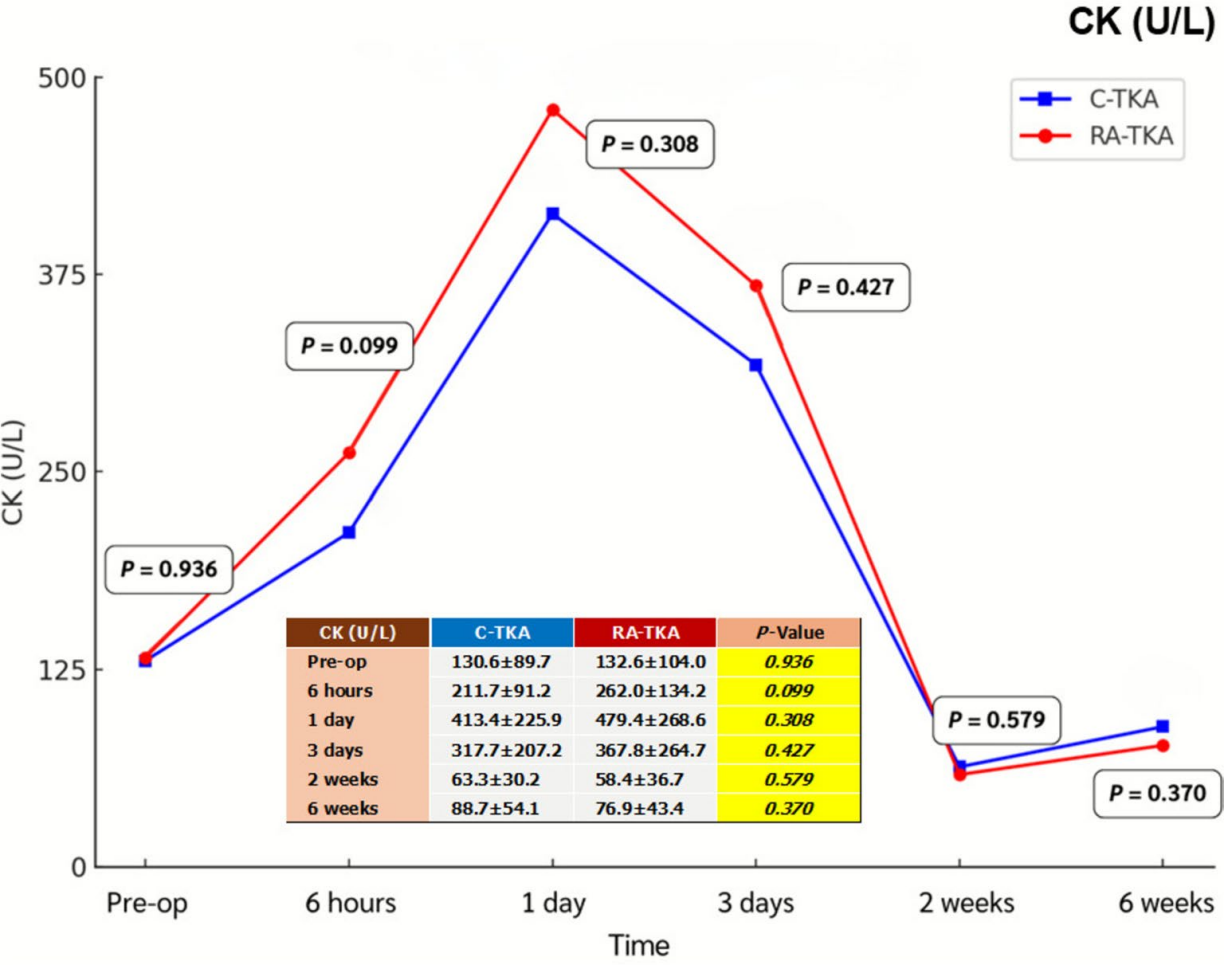
Comparison of systemic inflammatory biomarkers between C-TKA and RA-TKA in terms of creatine kinase (CK) levels at preoperative and postoperative time intervals. *C-TKA* conventional total knee arthroplasty, *RA-TKA* robotic-assisted total knee arthroplasty

**Fig. 6:**
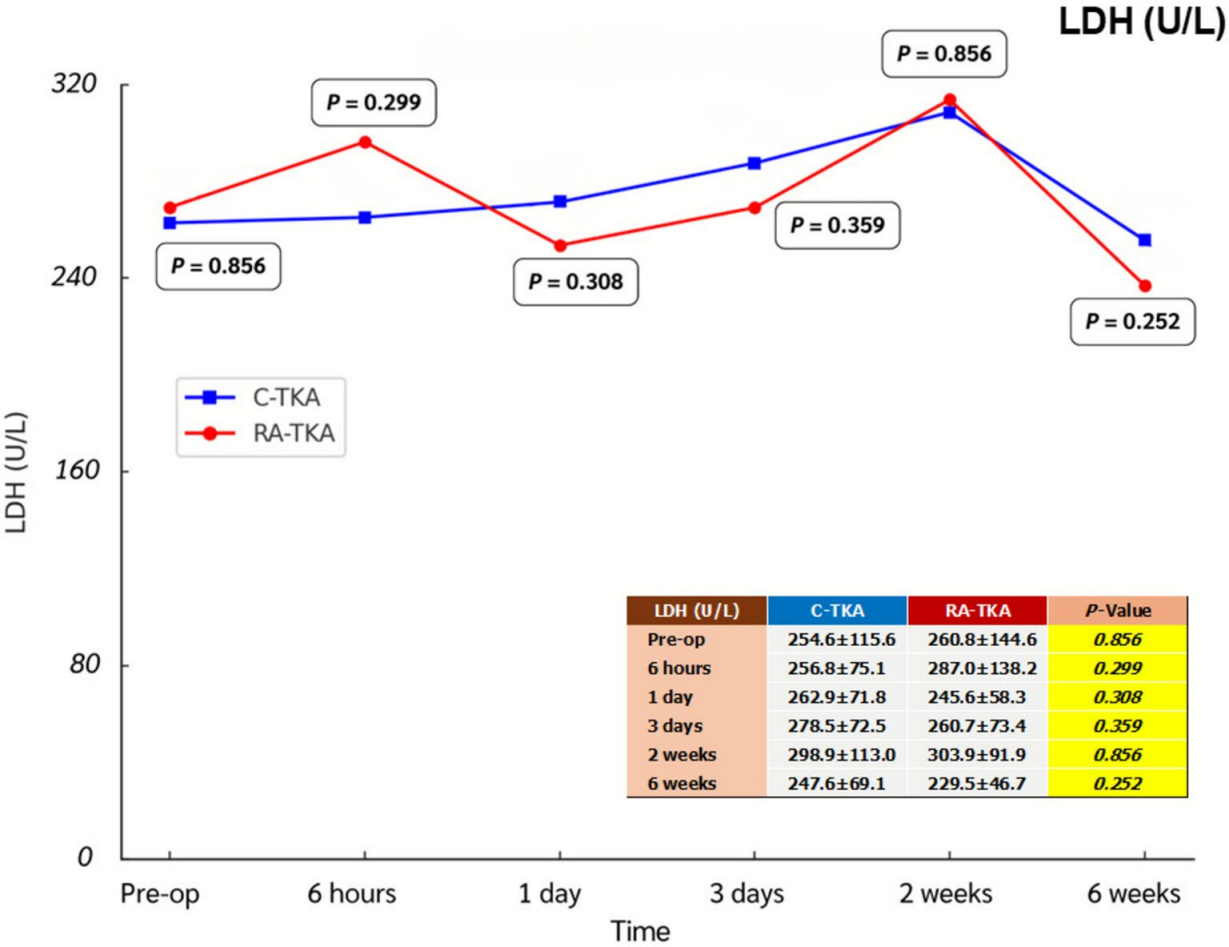
Comparison of systemic inflammatory biomarkers between C-TKA and RA-TKA in terms of lactate dehydrogenase (LDH) levels at preoperative and postoperative time intervals. *C-TKA* conventional total knee arthroplasty, *RA-TKA* robotic-assisted total knee arthroplasty

Serum IL-6, ESR, CRP, CK, and LDH levels showed no significant intergroup differences at any postoperative time point (6 h, 1 day, 3 days, 2 weeks, 6 weeks) (Table 2; Figs. 2, 3, 4, 5, 6). Although no statistically significant differences were observed, the between-group effect sizes for all biomarkers were small, indicating minimal clinical or biological differences.

**Table 2:** Serum systemic inflammatory biomarkers

Biomarkers^a^	Preoperative			6 h			1 day		
	C-TKA	RA-TKA	*p*-Value	C-TKA	RA-TKA	*p*-Value	C-TKA	RA-TKA	*p*-Value
IL-6 (pg/mL)	2.76 ± 2.12	2.38 ± 1.58	0.437	15.34 ± 7.80	14.58 ± 8.74	0.727	29.65 ± 30.08	42.19 ± 48.35	0.232
ESR (mm/h)	27.2 ± 13.0	29.7 ± 17.9	0.545	20.6 ± 13.3	22.1 ± 18.1	0.710	20.9 ± 12.4	22.1 ± 14.8	0.714
CRP (mg/L)	1.78 ± 1.88	1.32 ± 1.12	0.248	2.95 ± 3.65	1.56 ± 1.56	0.059	16.95 ± 14.89	16.81 ± 13.57	0.747
CK (U/L)	130.6 ± 89.7	132.6 ± 104.0	0.936	211.7 ± 91.2	262.0 ± 134.2	0.099	413.4 ± 225.9	479.4 ± 268.6	0.308
LDH (U/L)	254.6 ± 115.6	260.8 ± 144.6	0.856	256.8 ± 75.1	287.0 ± 138.2	0.299	262.9 ± 71.8	245.6 ± 58.3	0.308

Biomarkers^a^	3 days			2 weeks			6 weeks		
	C-TKA	RA-TKA	*p*-Value	C-TKA	RA-TKA	*p*-Value	C-TKA	RA-TKA	*p*-Value
IL-6 (pg/mL)	16.69 ± 16.58	15.71 ± 12.60	0.801	6.55 ± 5.20	5.39 ± 4.63	0.379	4.54 ± 3.88	4.21 ± 4.78	0.773
ESR (mm/h)	35.0 ± 19.1	36.7 ± 17.7	0.734	41.8 ± 16.9	44.9 ± 21.4	0.548	35.7 ± 16.7	37.5 ± 23.8	0.750
CRP (mg/L)	35.73 ± 55.21	29.08 ± 29.15	0.566	13.39 ± 17.58	7.53 ± 9.17	0.122	3.06 ± 2.31	2.66 ± 2.44	0.523
CK (U/L)	317.7 ± 207.2	367.8 ± 264.7	0.427	63.3 ± 30.2	58.4 ± 36.7	0.579	88.7 ± 54.1	76.9 ± 43.4	0.370
LDH (U/L)	278.5 ± 72.5	260.7 ± 73.4	0.359	298.9 ± 113.0	303.9 ± 91.9	0.856	247.6 ± 69.1	229.5 ± 46.7	0.252

^a^Independent *t*-test

All values are presented as mean ± standard deviation

*C-TKA* conventional total knee arthroplasty, *RA-TKA* robotic-assisted total knee arthroplasty, *IL-6* interleukin-6, *ESR* erythrocyte sedimentation rate, *CRP* C-reactive protein, *CK* creatine kinase, *LDH* lactic dehydrogenase

### Perioperative parameters

C-TKA had a significantly shorter tourniquet time than RA-TKA (Table 3).

**Table 3:** Perioperative outcomes

Characteristics^a^	RA-TKA (*n* = 30)	C-TKA (*n* = 30)	*p*-Value
Postoperative hematocrit (%)	36.6 ± 3.8	36.9 ± 3.3	0.729
Tourniquet time (min)	121.4 ± 15.3	95.0 ± 13.3	< 0.001^*^
Calculated estimated blood loss (mL)	247.0 ± 239.2	265.3 ± 207.5	0.753

All values are presented as mean ± standard deviation

^a^Independent *t*-test

^*^Indicates significant difference (*p* < 0.05)

Postoperative hematocrit and estimated blood loss did not differ significantly between groups. No patient required transfusion. Effect sizes for perioperative variables were similarly small, supporting the lack of meaningful intergroup differences.

### Radiographic evaluation

RA-TKA achieved superior alignment accuracy and fewer outliers compared with C-TKA (Tables 4, 5). The large effect size for mechanical alignment deviation demonstrates a substantial radiographic advantage of RA-TKA despite similar systemic biomarker responses.

**Table 4:** Postoperative radiographic results

Radiographic parameters^a^	RA-TKA (*n* = 30)	C-TKA (*n* = 30)	*p*-Value
Hip–knee–ankle angle (°)	0.3 ± 2.4	2.8 ± 3.4	0.002^*^
Lateral distal femoral angle (°)	91.0 ± 1.9	92.4 ± 2.8	0.030^*^
Medial proximal tibial angle (°)	90.8 ± 1.5	89.9 ± 2.1	0.047^*^
Tibiofemoral angle (°)	5.4 ± 2.0	3.2 ± 2.8	0.001^*^
Sagittal femoral component angle (°)	3.4 ± 1.9	2.7 ± 2.1	0.192
Sagittal tibial component angle (°)	1.8 ± 1.4	5.0 ± 2.6	< 0.001^*^

All values are presented as mean ± standard deviation

^a^Independent *t*-test

^*^Indicates significant difference (*p* < 0.05)

**Table 5:** Postoperative radiographic results and the number of knees with the implant aligned out of ± 3° from the target angle

Angle measured^a^	Knees with implant aligned out of ± 3° from the target angle (outlier)		
	RA-TKA (*n* = 30)	C-TKA (*n* = 30)	*p*-Value
Hip–knee–ankle angle	5 (17.9)	19 (65.5)	< 0.001^*^
Coronal femoral component angle	4 (14.3)	17 (58.6)	< 0.001^*^
Coronal tibial component angle	3 (10.7)	7 (24.1)	0.183
Sagittal femoral component angle	3 (10.7)	3 (10.3)	1.000
Sagittal tibial component angle	0 (0)	10 (34.5)	< 0.001^*^

All values are presented as *n* (%)

^a^Chi-squared test

^*^Indicates significant difference (*p* < 0.05)

### Clinical outcomes

Postoperative visual analog scale (VAS) pain scores were comparable between groups (Table 6). No significant difference in postoperative KSS was observed between RA-TKA and C-TKA (Table 6).

**Table 6:** Postoperative clinical outcomes

Postoperative clinical outcomes^a^	RA-TKA (*n* = 30)	C-TKA (*n* = 30)	*p*-Value
Visual analog scale score	1.3 ± 1.5	1.6 ± 1.9	0.508
Postoperative Knee Society Score			
Knee score	51.3 ± 13.8	46.1 ± 10.8	0.114
Functional score	77.6 ± 9.8	75.2 ± 13.8	0.448
Patient satisfaction score	34.9 ± 5.3	36.2 ± 5.0	0.350
Patient expectation score	13.2 ± 2.6	14.0 ± 2.2	0.240

All values are presented as mean ± standard deviation

^a^Independent *t*-test

## Discussion

This prospective randomized controlled trial found no significant differences in postoperative IL-6, ESR, CRP, CK, or LDH levels between RA-TKA and C-TKA, with uniformly small effect sizes indicating negligible biological differences.

Previous studies have reported mixed findings regarding the biological benefits of robotic systems. Some demonstrated transient reductions in early postoperative inflammatory markers [14–17], including lower IL-6, ESR, CRP, CK, and LDH in RA-TKA as shown by Kayani et al. [15] and early IL-6 and CK reductions reported by Xu et al. [14], whereas others observed no meaningful biomarker differences [25]. In our study, despite the use of a bone-milling technique specifically designed to minimize soft-tissue insult, systemic inflammatory biomarkers remained comparable, suggesting that biological stress from surgical exposure, soft-tissue handling, and component implantation may outweigh any differences in bone preparation methods.

Although bone-milling is theoretically advantageous, current evidence does not clearly support the burr as an independent factor reducing soft-tissue trauma. Prior studies indicate that reduced tissue injury in robotic TKA is more attributable to controlled implant positioning and fewer soft-tissue releases rather than to the bone-milling mechanism itself [18–21]. The similar biomarker profiles observed here likely reflect the dominant influence of global surgical factors, exposure, periarticular handling, component implantation, and tourniquet use over bone preparation technique. Furthermore, prior studies on inflammatory biomarkers in RA-TKA have largely involved saw-blade platforms, whereas the present study examined a milling-based robotic system. Because milling and saw-blade mechanisms may differ in their interaction with periarticular soft tissues, the limited soft-tissue advantage observed here may reflect platform-specific characteristics rather than inconsistencies with previous findings.

The longer operative time observed in this study should be interpreted in the context of the CORI workflow, which requires additional registration and verification steps. This platform-specific characteristic, rather than an inherent disadvantage of robotic TKA, likely accounted for the prolonged duration, as previous studies have shown minimal time differences once the learning curve is achieved [9]. The prolonged tourniquet duration inherent to the CORI workflow may also have contributed to postoperative inflammatory activation and potentially masked any biological advantage expected from bone-milling RA-TKA, consistent with the known relationship between operative duration and inflammatory response.

In addition, the operating surgeon was in the early phase of the robotic learning curve, having performed fewer than 50 RA-TKA cases at the initiation of this study. Importantly, no patients or cases were excluded from the analysis on the basis of operative or tourniquet time, and all randomized participants were analyzed according to the original study protocol. As presented in Table 3, RA-TKA was associated with a significantly longer tourniquet time than C-TKA; this finding is best interpreted in the context of the early learning phase of the robotic platform rather than selective case exclusion or protocol deviation. Previous studies have demonstrated that operative and tourniquet times decrease substantially after approximately 20–50 cases and are no longer a major disadvantage of RA-TKA once sufficient experience is achieved [26].

In contrast, RA-TKA demonstrated superior radiographic accuracy, aligning with prior evidence showing fewer alignment outliers and improved mechanical precision [6, 9, 25]. Despite longer operative time, estimated blood loss and early functional outcomes were similar between groups, indicating that the primary advantage of bone-milling RA-TKA lies in mechanical reproducibility rather than measurable reductions in systemic inflammation.

### Limitations

First, RA-TKA requires the placement of additional registration pins, resulting in extra wound closure, and blinding of the surgeon and patients was not feasible owing to differences in surgical techniques. Second, although soft-tissue injury was assessed using systemic inflammatory biomarkers, no direct intraoperative macroscopic soft-tissue evaluation was performed. A validated grading system, such as the macroscopic soft-tissue injury (MASTI) score [27], was not prospectively collected, limiting assessment to indirect measures, including serum biomarkers, tourniquet time, estimated blood loss, and clinical outcomes. The absence of an objective macroscopic soft-tissue grading system is a major limitation, as systemic biomarkers may not fully reflect localized periarticular trauma. Third, the extent of periarticular soft-tissue release was not systematically documented, preventing quantitative comparison between groups and representing an additional limitation of the study. Fourth, systemic inflammatory markers may be influenced by confounders such as comorbidities, incision length, tourniquet duration, and postoperative activity levels, all of which may affect biomarker interpretation. Additionally, the lack of intraarticular fluid analysis limits detection of localized inflammatory responses; integrating both local and systemic biomarkers may yield a more comprehensive biological assessment. Finally, the relatively short follow-up period limits evaluation of long-term outcomes, including late complications and revision rates. Longer-term investigations are warranted to determine whether the mechanical advantages of robotic bone-milling TKA translate into sustained clinical or biological benefits.

## Conclusions

Bone-milling RA-TKA did not demonstrate significant differences in systemic inflammatory biomarkers compared with C-TKA, indicating a limited impact on soft-tissue preservation. However, it consistently achieved superior radiographic alignment accuracy. The longer operative duration observed in the RA-TKA group reflects the early learning phase and did not translate into differences in estimated blood loss or early postoperative recovery.

### Clinical implication and future direction

Bone-milling RA-TKA should be regarded primarily as a precision-enhancing surgical technology rather than a biologically protective procedure. Its adoption may be best justified in scenarios where alignment accuracy and reproducibility are paramount, such as in complex deformities or revision settings. Future research incorporating local tissue-level inflammatory assessments and long-term functional follow-up is warranted to determine whether improved radiographic precision ultimately translates into enhanced implant longevity and patient satisfaction.

## Data Availability

The datasets generated during and/or analyzed during the current study are available from the corresponding author on reasonable request.
